# Assembly of One-Patch Colloids into Clusters via Emulsion Droplet Evaporation

**DOI:** 10.3390/ma10040361

**Published:** 2017-03-29

**Authors:** Hai Pham Van, Andrea Fortini, Matthias Schmidt

**Affiliations:** 1 Theoretische Physik II, Physikalisches Institut, Universität Bayreuth, Universitätsstraße 30, D-95440 Bayreuth, Germany; 2 Department of Physics, Hanoi National University of Education, 136 Xuanthuy, Hanoi 100000, Vietnam; haipv@hnue.edu.vn; 3 Department of Physics, University of Surrey, Guildford GU2 7XH, UK; a.fortini@surrey.ac.uk

**Keywords:** one-patch colloid, Janus colloid, cluster, emulsion-assisted assembly, Pickering effect

## Abstract

We study the cluster structures of one-patch colloidal particles generated by droplet evaporation using Monte Carlo simulations. The addition of anisotropic patch–patch interaction between the colloids produces different cluster configurations. We find a well-defined category of sphere packing structures that minimize the second moment of mass distribution when the attractive surface coverage of the colloids *χ* is larger than 0.3. For *χ* < 0.3, the uniqueness of the packing structures is lost, and several different isomers are found. A further decrease of *χ* below 0.2 leads to formation of many isomeric structures with less dense packings. Our results could provide an explanation of the occurrence of uncommon cluster configurations in the literature observed experimentally through evaporation-driven assembly.

## 1. Introduction

Packings of colloidal particles in regular structures are of great interest in colloid science. One particular class of such packings is formed by colloidal clusters which can be regarded as colloidal analogues to small molecules, i.e., “colloidal molecules” [1,2]. In pioneering work, Manoharan, Elsesser, and Pine [3] reported a method for the fabrication of clusters of microspheres. The microspheres absorb at the interface of (liquid) emulsion droplets. During the droplet evaporation, capillary forces and van der Waals attractive interactions pack the micropheres into stable clusters. The final cluster configurations are unique (i.e., possess a single well-defined geometry) for $n_{c}⩽15$ with $n_{c}$ the number of constituent particles. Lauga and Brenner [4] later have shown that the unique configuration of each $n_{c}$-sphere cluster can be correctly predicted by minimization of the total surface energy of the droplet–particle interface. Remarkably, these configurations with $n_{c}⩽11$ exactly correspond to those obtained from the calculation of the packings that minimize the second moment of the mass distribution, namely $M_{2}$-minimal packings ($M_{2}$-minimal clusters), by Sloane et al. [5]. In addition to $M_{2}$-minimal packings, many other optimal sphere packings based on optimization principles have been investigated, such as the Lennard–Jones packing problem [6], Coulomb packing [7], spherical packing [8] and hard-sphere packing with a short-range attraction [9]. The geometric structures of the Lennard–Jones packing were obtained by mimimizing the Lennard–Jones potential between spheres. Coulomb packing, sometimes known as the Thomson problem [7], is an equilibrium arrangement of identical point charges on a sphere so that the total electrostatic potential energy is minimal. In the spherical packing or the Tammes problem [8], monodisperse hard spheres arrange on the surface of another sphere such that the smallest distance between the center-center distance of spheres is maximized, or equivalently maximizing the area density of spheres. Interestingly, all these packings have exactly the same structures for $n_{c}$ up to six, but differ at most values $n_{c}>6$.

A higher level of complexity of cluster structures can be achieved when the colloidal shape is extended beyond spherical. Peng and cowokers [10] prepared clusters of dumbbell-shaped particles and indicated that minimization of $M_{2}$ is not a general rule for aggregation of shape-anisotropic particles. Open clusters with a compact core and protruding arms were obtained by varying the relative size of colloidal spheres in dumbbells [11]. Besides extending the colloidal shape, anisotropic surface chemistry has attracted considerable attention [12]. Granick et al. [13] prepared clusters of charged Janus particles, i.e., spherical particles possessing oppositely charged hemispheres. The authors later considered clusters of amphiphilic Janus colloids whose two hemispheres are negatively charged and hydrophobic, respectively [14,15]. Experimental results that were complemented by Monte Carlo simulations revealed the existence of several cluster structures that belong to both the conventional polyhedra and less densely packed structures. However, the authors investigated cluster formation without emulsion droplets, and therefore the resulting clusters may represent intermediate states of larger packings because there is no limitation to the size of clusters that can form through electrostatic interactions. Sciortino and coworkers performed numerical simulations to investigate the collective structure as well as the phase behavior of one-patch particles [16,17]. They found that self-assembly of such colloids gives rise to a rich variety of increasingly complex structures and produces unconventional phase equilibria.

Despite the number of experimental [18,19,20], theoretical [21,22,23] and simulation works [16,17,23] on assemblies of one-patch particles, there is no report about cluster assembly through emulsion droplet evaporation of colloidal particles with such anisotropic interparticle potentials. In this article, we study a binary mixture of one-patch colloidal particles and emulsion droplets. To characterize the anisotropic pair interactions between patches on two colloid surfaces, we employ a simple model proposed by Kern and Frenkel [24]. An advantage of the Kern–Frenkel potential is that the competing short-ranged repulsive and attractive interactions between patches can be tuned by simply changing the surface coverage. Similar to previous work [25], we use Monte Carlo simulations to simulate the dynamic pathways of cluster formation. We find the cluster structures to be in good agreement with the majority of experimental structures in the literature. This finding also indicates that strong short-ranged repulsive interactions between colloidal spheres result in the occurrence of particular cluster structures in addition to common $M_{2}$-minimal clusters.

This article is organized as follows. In Section 2, we give the details of the pair interactions and simulation method. In Section 3, we compare the final cluster structures, histograms of cluster size distribution for different values of the surface coverages. Additionally, we analyze the dynamics of cluster formation and an orientational order parameter of the final clusters. Conclusions are given in Section 4.

## 2. Model and Methods

**Figure 1:**
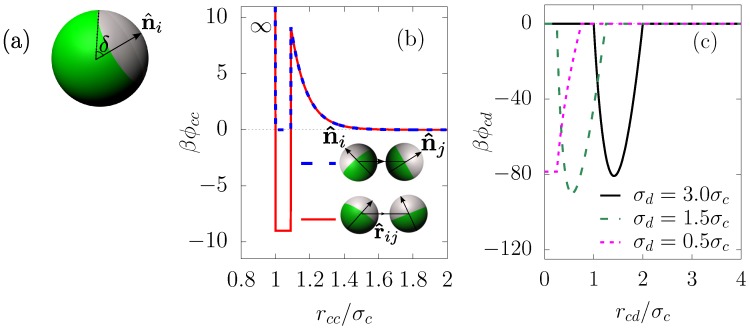
Pair potentials for the binary mixture of patchy colloids and droplets. (**a**) illustration of a single patchy colloid *i* with orientation ${\hat{n}}_{i}$ and opening angle *δ* of the attractive patch (white); (**b**) potentials between two colloids with $\kappa \sigma_{c}=10$, $\beta \epsilon_{Y}=24.6$, $\beta \epsilon_{\mathrm{SW}}=9$, $\Delta =0.09\sigma_{c}$ $\beta =1/k_{B}T$. Shown in the legend is the Janus case χ=1/2. When the attractive parts of two particles properly face each other, they interact via the square-well potential of depth $18k_{B}T$ (red solid line), and otherwise they interact via the square-well potential of depth $9k_{B}T$ (blue dashed line); (**c**) colloid–droplet potential at $\sigma_{d}\left(t\right)/\sigma_{c}=3$, 1.5 and 0.5.

We study a binary mixture of $N_{c}$ one-patch colloids with hard-sphere diameter $\sigma_{c}$ and $N_{d}$ spherical droplets of diameter $\sigma_{d}$. Each colloid possesses a central position and a unit vector ${\hat{n}}_{i}$ locating the direction of patch on the *i*th particle surface. The size of the attractive patch is determined by a conical segment of (half) opening angle *δ* around the direction ${\hat{n}}_{i}$ (Figure 1a). It is convenient to define the surface coverage *χ* as the relative ratio between the attractive surface area and total surface area. Therefore, *χ* is related to the half opening angle *δ* via

(1) $$\begin{array}{c} \chi =\sin^{2}\left(\frac{\delta }{2}\right). \end{array}$$

The patch–patch interaction between colloid *i* and *j* is described by a Kern–Frenkel (KF) potential, $U_{\mathrm{KF}}\left({\hat{r}}_{ij},{\hat{n}}_{i},{\hat{n}}_{j}\right)$, defined as a product of a square-well (SW) potential with an angular modulation

(2) $$\begin{array}{c} U_{\mathrm{KF}}\left(r_{ij},{\hat{n}}_{i},{\hat{n}}_{j}\right)=U_{\mathrm{SW}}\left(r\right)\Psi \left({\hat{r}}_{ij},{\hat{n}}_{i},{\hat{n}}_{j}\right), \end{array}$$

where

(3) $$\begin{array}{c} U_{\mathrm{SW}}\left(r\right)=\left\{\begin{array}{cc} \infty , & r<\sigma_{c},\\ -\epsilon_{\mathrm{SW}}, & \sigma_{c}<r<\sigma_{c}+\Delta ,\\ 0, & \mathrm{otherwise}, \end{array}\right. \end{array}$$

and

(4) $$\begin{array}{c} \Psi \left({\hat{r}}_{ij},{\hat{n}}_{i},{\hat{n}}_{j}\right)=\left\{\begin{array}{cc} 1, & \mathrm{if}\left\{\begin{array}{cc} & {\hat{n}}_{i}\cdot {\hat{r}}_{ij}⩾\cos\delta ,\\ \mathrm{and} & -{\hat{n}}_{j}\cdot {\hat{r}}_{ij}⩾\cos\delta , \end{array}\right.\\ 0, & \mathrm{otherwise}, \end{array}\right. \end{array}$$

where $U_{\mathrm{SW}}\left(r\right)$ is an isotropic square-well potential of depth $\epsilon_{\mathrm{SW}}$ and width Δ. $\Psi ({\hat{r}}_{ij},{\hat{n}}_{i},{\hat{n}}_{j})$ is a modulation function that depends on the relative orientation of the two particles, ${\hat{n}}_{i}$ (${\hat{n}}_{j}$) is the unit vector pointing from the center of sphere *i* (*j*) to the center of the corresponding attractive patch and ${\hat{r}}_{ij}=r_{ij}/\left|r_{ij}\right|$ is the unit vector of distance between the centers of two spheres *i* and *j*.

By varying the surface coverage *χ*, one can control the angular range of the anisotropic interaction. The special case of χ=1/2 ($\delta =90^{\circ }$) is known as the Janus limit with half–half geometry. In the extreme case χ=0, the pair potential reduces to a hard-core interaction, while, for χ=1, it reduces to an isotropic square-well interaction.

The Yukawa repulsion $U_{Y}\left(r\right)$ describes the interaction between two charged colloidal particles screened by a electrolyte solution with inverse Debye length *κ*, i.e.,

(5) $$\begin{array}{c} U_{Y}\left(r\right)=\epsilon_{Y}\sigma_{c}\frac{\exp[-\kappa \left(r-\sigma_{c}\right)]}{r}, \end{array}$$

where the parameter $\epsilon_{Y}$ controls the strength of the long-ranged repulsion.

The colloid–colloid pair interaction, $\phi_{cc}\left(r_{ij},{\hat{n}}_{i},{\hat{n}}_{j}\right)$, is expressed in terms of the anisotropic, short-ranged attraction $U_{\mathrm{KF}}\left({\hat{r}}_{ij},{\hat{n}}_{i},{\hat{n}}_{j}\right)$ and the longer-ranged Yukawa repulsion $U_{Y}\left(r\right)$, i.e.,

(6) $$\begin{array}{c} \phi_{cc}\left(r_{ij},{\hat{n}}_{i},{\hat{n}}_{j}\right)=\left\{\begin{array}{cc} \infty , & r<\sigma_{c},\\ U_{\mathrm{KF}}\left(r\right), & \sigma_{c}<r<\sigma_{c}+\Delta ,\\ U_{Y}\left(r\right), & \mathrm{otherwise}. \end{array}\right. \end{array}$$

As illustrated in Figure 1b, the potential $\phi_{cc}\left({\hat{r}}_{ij},{\hat{n}}_{i},{\hat{n}}_{j}\right)$ is plotted for a typical set of parameters (justified below). Here, two colloids interact via the square-well potential of depth $9k_{B}T$ or $18k_{B}T$ depending on the orientation of the patch unit vectors ${\hat{n}}_{i}$ and ${\hat{n}}_{j}$; and the distance between the two particles is within the range $\left(\sigma_{c},\sigma_{c}+\Delta \right)$. Note that we choose a sufficiently large strength of the attractive interaction ($9k_{B}T-18k_{B}T$) to ensure that physical bonds between colloids once formed via droplet evaporation are permanent. The repulsive barrier is also set to be large enough in order to hinder spontaneous clustering, i.e., clustering that is not mediated by the droplets. In principle, one can choose a wide range of interacting parameters subject to the two above constraints without qualitatively affecting the final results.

The droplet–droplet interaction is aimed at modeling the repulsive interaction of charged droplets so that coalescence is negligible. Furthermore, in order to avoid the binding between any two droplets due to a shared Janus colloid, we assume that each droplet has an effective interaction diameter $\sigma_{d}+\sigma_{c}$ that is larger than the geometric droplet diameter $\sigma_{d}$. Hence, the droplet–droplet pair interaction is

(7) $$\begin{array}{c} \phi_{dd}\left(r\right)=\left\{\begin{array}{cc} \infty , & r<\sigma_{d}+\sigma_{c},\\ 0, & \mathrm{otherwise}. \end{array}\right. \end{array}$$

Similarly to the previous model of Schwarz et al. [25], we assume that the colloid–solvent interfacial tension is equal to the colloid–droplet interfacial tension, so that the contact angle is 90°. This assumption is reasonable since a change in the contact angle seems to not have an influence on the final outcomes [4]. We neglect the influence of the adsorbed colloids on the droplet shape and hence assume that the droplets remain spherical. We also assume that the colloid–droplet interaction is isotropic. In reality, Janus particles will in general have differing wetting properties of their two types of surfaces. Therefore, one would expect preferential orientation of a colloid that is adsorbed on a droplet surface. Within our current model, we neglect this effect and restrict ourselves to colloids with identical wetting properties. More complex investigations [26,27] would be necessary to describe orientational effects.

The evaporation of the dispersed oil droplet implies that the droplet diameter is initially larger and eventually smaller than the colloid diameter. In order to mimic this situation, the colloid–droplet potential $\phi_{cd}\left(r\right)$ is given as follows:

If $\sigma_{d}>\sigma_{c}$,

(8) $$\begin{array}{c} \phi_{cd}\left(r\right)=\left\{\begin{array}{cc} -\gamma \pi \sigma_{d}h & \frac{\sigma_{d}-\sigma_{c}}{2}<r<\frac{\sigma_{d}+\sigma_{c}}{2},\\ 0, & \mathrm{otherwise}, \end{array}\right. \end{array}$$

and if $\sigma_{d}<\sigma_{c}$,

(9) $$\begin{array}{c} \phi_{cd}\left(r\right)=\left\{\begin{array}{cc} -\gamma \pi \sigma_{d}^{2} & r<\frac{\sigma_{c}-\sigma_{d}}{2},\\ -\gamma \pi \sigma_{d}h & \frac{\sigma_{c}-\sigma_{d}}{2}<r<\frac{\sigma_{c}+\sigma_{d}}{2},\\ 0, & \mathrm{otherwise}, \end{array}\right. \end{array}$$

with *γ* the colloid–droplet interfacial tension and *h* the height of the spherical cap that results from the colloid–droplet intersection given by

(10) $$\begin{array}{c} h=\frac{\left(\sigma_{c}/2-\sigma_{d}/2+r\right)\left(\sigma_{c}/2+\sigma_{d}/2-r\right)}{2r}. \end{array}$$

Figure 1c shows the colloid–droplet pair potential as a function of the scaled distance for several different ratios of the droplet diameter and colloid diameter.

The total interaction energy *U* is written as the sum of colloid–colloid, droplet–droplet, and colloid–droplet pair interactions,

(11) $$\begin{array}{c} \begin{array}{ccc} \frac{U}{k_{B}T} & = & \sum_{i<j}^{N_{c}}\phi_{cc}\left(r_{ij},{\hat{n}}_{i},{\hat{n}}_{j}\right)+\sum_{i<j}^{N_{d}}\phi_{dd}\left(\right|R_{i}-R_{j}\left|\right)\\ & & +\sum_{i}^{N_{c}}\sum_{j}^{N_{d}}\phi_{cd}\left(\right|r_{i}-R_{j}\left|\right), \end{array} \end{array}$$

where $\left(r_{i},{\hat{n}}_{i}\right)$ and $\left(r_{j},{\hat{n}}_{j}\right)$ is the center-of-mass coordinate and the unit vector locating the attractive patch of colloid *i* and colloid *j*, respectively; $R_{i}$ is the center-of-mass coordinate of droplet *i*, $k_{B}$ is the Boltzmann constant, and *T* is the temperature.

The binary mixture of patchy colloids and droplets was simulated in the canonical ensemble using the kinetic Monte Carlo (MC) method. The total number of MC cycles per particle is $10^{6}$ with $5\times 10^{5}$ MC cycles used for droplet shrinkage and the remaining $5\times 10^{5}$ MC cycles used to equilibrate the simulation system. In each MC cycle, we attempt to move each particle once on average. A maximum trial displacement $d_{c}$ and maximum rotation step of the colloids $\theta_{c}$ are set to $d_{c}=0.01\sigma_{c}$ and $\theta_{c}=0.01\mathrm{rad}$, respectively. The droplets move with a maximum trial displacement $d_{d}=d_{c}\sqrt{\sigma_{c}/\sigma_{d}}$ and shrink at a constant rate such that their diameter vanishes completely after $5\times 10^{5}$ MC cycles. The choice of such small movement steps enables to approximate the Brownian dynamics [28]. The physical time can be expressed in terms of the total number of MC cycles per particle. From the Einstein relation and the Stokes–Einstein equation for diffusion of spherical particles, we roughly estimate the physical time of the evaporation process to be on the order of seconds. For details of the calculation, see reference [11]. Although this physical time is quite small compared to experimental timescales that typically last tens of minutes, we do not expect the MC timescale to affect the final outcomes [25]. In addition, within the kinetic MC simulation, we perform sequential moves of individual particles and neglect the collective motion of particles in the cluster, i.e., collective translational and rotational cluster moves are not carried out. Such collective modes of motion only play a role in dense colloidal systems with interpaticle attractive interactions that vary strongly with distance or angle [29,30].

All simulations are performed in a cubic box with periodic boundary conditions for the binary mixture of $N_{c}=500$, $N_{d}=12$, at a fixed colloid packing fraction $\eta_{c}=0.03$, and droplet packing fraction $\eta_{d}=0.15$. The initial droplet diameter $\sigma_{d}\left(0\right)$ is set to $6\sigma_{c}$. We have studied one-patch colloids for different values of attractive patches, i.e., *χ* between 0 and 1, by varying the value of cosδ. For a given set of parameters, statistical data is collected by running 20 independent simulations.

We determine the existence of a bond between two colloids when their distance is smaller than $\sigma_{c}+\Delta$ and define a cluster as a network of colloids that are connected with each other by bonds. Each cluster (isomer) is therefore described by both the number of colloids $n_{c}$ and the number of bonds $n_{b}$. To initialize the simulation, the colloids are distributed randomly outside droplets, i.e., $r_{cd}>\left[\sigma_{c}+\sigma_{d}\left(0\right)\right]/2$ and with random orientations. The minimum distance between the colloids is set larger than one bond length $\sigma_{c}+\Delta$. In this way, no two colloids form a bond in the initial stage of the simulation.

## 3. Results and Discussion

**Figure 2:**
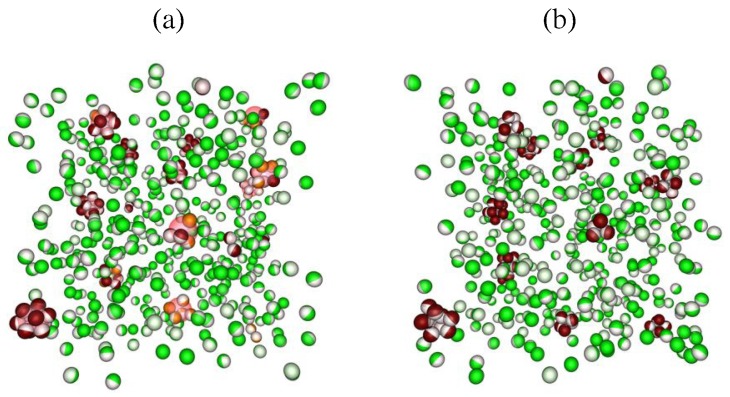
Simulation snapshots of the mixture for Janus colloids (χ=1/2) and droplets. Results are shown at two different stages of the time evolution: (**a**) after $3.25\times 10^{5}$ MC cycles and (**b**) after $10^{6}$ MC cycles. Droplets are depicted as pink spheres. Each Janus colloid has two hemispheres where the white hemisphere corresponds to the attractive patch, and the green one is the repulsive patch. When colloidal particles are trapped at the droplet surface or in droplet-induced clusters, the repulsive part of Janus colloids are shown in red.

Figure 2 shows snapshots at two different stages of the time evolution for the colloid surface coverage χ=1/2. After $3.25\times 10^{5}$ MC cycles (Figure 2a), the droplets (pink spheres) capture several Janus colloids (green-white spheres) and pull them into clusters (red-white spheres). Figure 2b shows the final configuration after $10^{6}$ MC cycles. All droplet-induced clusters have well-defined structures and are stable against thermal fluctuations on the time scales considered.

Compared to the simulation results obtained at an isotropic, short-ranged, attractive colloid–colloid pair potential [25], our model for the one-patch colloids reproduces similar cluster structures at surface coverages χ⩾0.30. Here, we find stable clusters of unique configurations, including dumbbell ($n_{c}=2$), triplet ($n_{c}=3$), tetrahedron ($n_{c}=4$), triangular dipyramid ($n_{c}=5$), octahedron ($n_{c}=6$), and pentagonal dipyramid ($n_{c}=7$). For higher order clusters ($n_{c}⩾8$), we find snub disphenoid ($n_{c}=8$), triaugmented triangular prism ($n_{c}=9$), gyroelongated square dipyramid ($n_{c}=10$), icosahedron minus one ($n_{c}=11$) and icosahedron ($n_{c}=12$). Clusters with $n_{c}=13$–15 are also found but skipped for analysis because of their multiple structures. These structures ($n_{c}=4$ to 12, except for $n_{c}=11$) belong to a set of convex polyhedra with equilateral triangular faces, known as convex deltahedra that minimize the second moment of the mass distribution [5], $M_{2}=\sum_{i=1}^{n_{c}}{\left|r_{i}-r_{\mathrm{cm}}\right|}^{2}$, where $r_{i}$ is the position of the particle *i* and $r_{\mathrm{cm}}$ is the position of the cluster center-of-mass. Notably, such structures satisfy the relation $n_{b}=3n_{c}-6$ and are found to be identical to those of colloidal clusters observed through evaporation-driven assembly [3,31].

**Figure 3:**
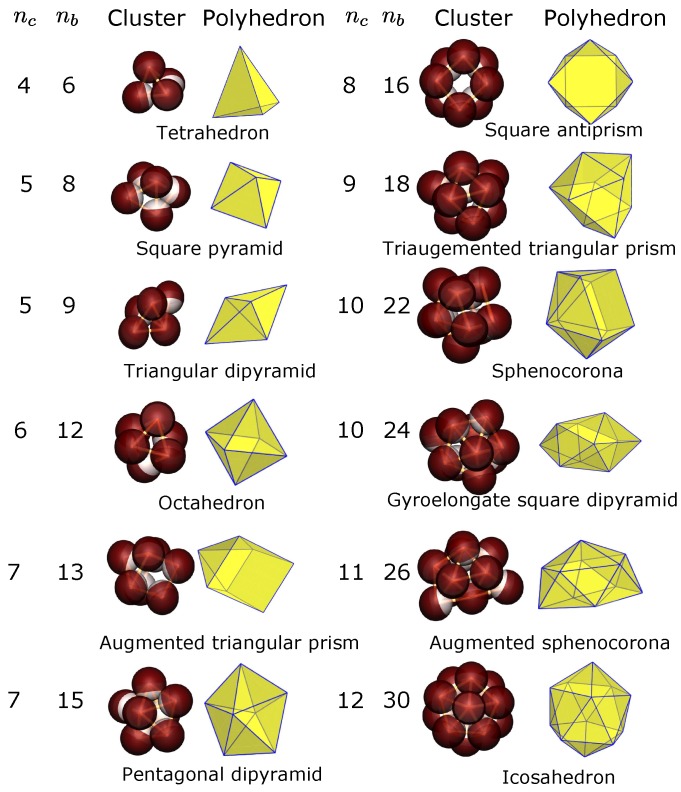
Typical cluster structures obtained in simulations (middle columns) at surface coverage χ=0.25. The white colloid patch is attractive and the red one is repulsive. The wire frame connecting the colloid centers illustrates the bond skeleton. Shown in the left columns are the number of constituent colloids $n_{c}$ and the number of bonds $n_{b}$ of the corresponding clusters. Right columns illustrate the polyhedra formed by drawing lines from the center of the colloidal sphere to its neighbors. Below are the names of the polyhedra from [32].

Figure 3 shows the cluster structures obtained at surface coverage χ=0.25. We find clusters containing 2 to 12 constituent spheres (dumbbells and triplets not shown). For $n_{c}=5$, 7, and 10, clusters of same $n_{c}$ show two different structures corresponding to two different bond numbers $n_{b}$, whereas for the clusters with the remaining colloid numbers $n_{c}$, only one specific structure is observed. We consider in turn each of the $n_{c}$-sphere clusters and adopt the convention that all clusters that do not minimize $M_{2}$ are referred to $M_{2}$-nonminimal clusters.

For clusters of five constituent spheres ($n_{c}=5$), two different isomers are found, i.e., an uncommon isomer of the square pyramidal structure (eight bonds) and an $M_{2}$-minimal isomer of the triangular dipyramid structure (nine bonds). In agreement with the results of Wittemann et al. [25,33], we find that the square pyramidal isomers possess a very small fraction of the total number of clusters compared to that of the triangular dipyramidal isomers. For $n_{c}=6$, only octahedral clusters are found. For the seventh order clusters, we find pentagonal dipyramidal clusters (15 bonds) together with clusters of augmented triangular prim configuration (13 bonds), both of which are found with the same probability. Interestingly, clusters with the square pyramid and augmented triangular prism structure were also observed through evaporation-driven assembly, such as clusters of silica particles from aerosol droplets [34], clusters of polystyrene particles coated with silica particles [35], and clusters of crossliked polystyrene/divinylbenzene microspheres mixed with polystyrene polymers [36]. For 8-sphere clusters, single-patch colloids assemble into the square antiprism instead of the $M_{2}$-minimal snub disphenoid. This result agrees well with that of the cluster sample obtained from aqueous aerosol droplets [34]. In addition, the square antiprism configuration was frequently found in experiments of Cho et al. [34,37,38] and other authors [33,36]. Cho and coworkers explained the square antiprism configuration, which matches the geometry of a Coulomb cluster for $n_{c}=8$ [7], as a result of the electrostatic repulsion between the particles in emulsion droplets. In our simulations, we find this configuration only when the attractive surface coverage *χ* is less than 0.3. In other words, choosing a sufficiently large, short-ranged, repulsive part of the Kern–Frenkel potential between the single-patchy colloids leads to the formation of this configuration. For $n_{c}=9$, the colloids pack into a specific configuration that is identical to the triaugemented triangular prism except for some missing bonds. Two specific structures are found again for clusters of order $n_{c}=10$; one is a cluster of gyroelongated square dipyramid and the other a sphenocorona. Remarkably, while the former structure is the familiar $M_{2}$-minimal cluster, the latter structure seems not to be a member of sphere packings generated by global minimal constraints [5,6,7,9] and have not yet been observed experimentally as well. In the case of the eleventh order clusters ($n_{c}=11$), differently from a $M_{2}$-minimal nonconvex structure in the experiments as discussed in the context, we find a convex augmented sphenocorona. To our knowledge, only one other study has detected the convex structure of the 11-sphere clusters [37]. Finally, for $n_{c}=12$, all colloids assemble into clusters with icosahedral symmetry.

For the same number of constituent colloids $n_{c}$, a common feature of the above $M_{2}$-nonminimal clusters is their smaller bond number compared to that of the $M_{2}$-minimal clusters. Each of the $M_{2}$-nonminimal polyhedra in Figure 3 has at least one square face instead of all triangular faces as in the case of the $M_{2}$-minimal polyhedra. For example, the square pyramid ($n_{c}=5$) contains one square face, the augmented triangular prism ($n_{c}=7$) and square antiprism ($n_{c}=8$) include two square faces. A second point to be made is that three configurations (tetrahedron, octahedron, and icosahedron) where all sphere positions are equivalent are found in the simulations, regardless of the value of *χ*. This result indicates that clusters with high symmetry appear to be insensitive to the interactions between particles.

**Figure 4:**
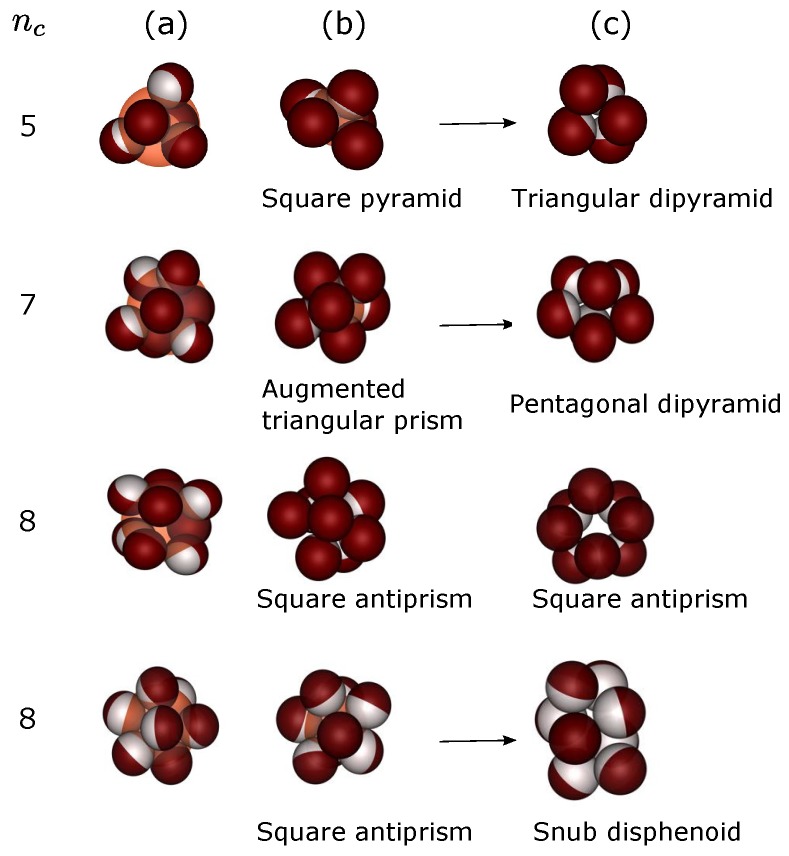
Simulation snapshots of $n_{c}$-sphere clusters of the packing process. The column (**a**) shows a droplet (shown in pink large sphere) and single-patch colloids trapped at its surface after $3.5\times 10^{5}$ MC cycles. After $4.5\times 10^{5}$ MC cycles (column (**b**)), the droplet has shrunk and the colloids have packed into a well-defined structure. In the final stage of the simulations (column (**c**)), some clusters are rearranged to form $M_{2}$-minimal clusters. The arrows refer to the structures that are rearranged during evaporation. The first rows are the results in the case of χ=0.25, and the last row is for χ=0.5.

We analyze the mechanism by which the clusters form using visual inspection. Figure 4 shows ‘time’-lapsed frames of the clusters for $n_{c}=5$, 7 and 8. In column (a), the colloids are bound to the droplet surface and freely diffusing at its surface. After $4.5\times 10^{5}$ MC cycles, the colloids are arranged as $M_{2}$-nonminimal packings (column (b)). Since then, two scenarios are possible for the clusters containing 5 and 7 colloids. In the first scenario the colloids are rearranged to form the final $M_{2}$-minimal packings with the higher bond number; in the second scenario (not shown), the final packings of the colloids remain $M_{2}$-nonminimal packings. However, for the 8-sphere cluster, only the second scenario appears. The colloids in this configuration show less motion in comparison with those of the cluster configurations with five and seven constituents. The weak attractive part between the colloids might prevent rearrangement in the 8-sphere cluster. This is evident in the last row of Figure 4, which shows the scenario of the 8-sphere cluster but for the surface coverage χ=0.5 or, equivalently, a higher attractive part. We observe a structural transformation from the square antiprism to the snub disphenoid configuration, which is not observed in the case of χ=0.25. Therefore, all $M_{2}$-minimal clusters may represent collapsed states of $M_{2}$-nonmimimal clusters.

**Figure 5:**
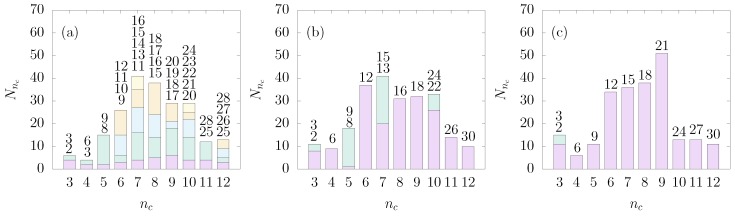
Distribution of the number of clusters $N_{n_{c}}$ with $n_{c}$ colloids at three different coverages: (**a**) χ=0.125; (**b**) χ=0.25; and (**c**) χ=0.5. Each differently colored bar is labeled, from top to bottom, with the bond number $n_{b}$.

Figure 5 shows a stacked histogram of the number of clusters $N_{n_{c}}$ with $n_{c}$ constituent colloids at three different values of *χ*. The height of each differently colored bar is proportional to the number of clusters with the bond number $n_{b}$. For a small value of *χ* (Figure 5a), a variety of different isomers with small bond numbers are observed. We interpret this as as a direct result of the difficult equilibration of cluster structures in the presence of a strong patch–patch repulsion. In addition, the distribution of the number of clusters shows a large fraction of the clusters with colloid numbers between six and ten. At a higher value of *χ* (χ=0.25), there is no significant change in the cluster size distribution. However, the number of distinct isomers decreases, as shown in Figure 5b. For the clusters with $n_{c}=3$, 5, 7 and 10, two isomers are possible, while for the clusters with the remaining values of $n_{c}$, only one isomer is present. When *χ* is larger than 0.30, e.g., χ=0.5, almost all clusters have only a single well-defined structure (see Figure 5c).

As the cluster configurations are in a stable state in the final stage of the simulations, the constituent colloids align in such a way that their attractive patches face each other in order to minimize the total potential energy. We employ an orientational order parameter that was used in a classification of self-assembled structures of patchy colloidal dumbbells [39], defined as

(12) $$\begin{array}{c} M=\langle \frac{1}{n_{c}}\sum_{i=1}^{n_{c}}{\hat{n}}_{i}\cdot \frac{r_{\mathrm{cm}}-r_{i}}{\left|r_{\mathrm{cm}}-r_{i}\right|}\rangle , \end{array}$$

where the angular brackets denote an average over all clusters that are composed of $n_{c}$ constituent colloids, $r_{i}$ is the center of mass of colloid *i*, $r_{\mathrm{cm}}$ is the center of mass of the cluster and ${\hat{n}}_{i}$ is the unit vector pointing in the direction of the attractive patch of colloid *i* (c.f. Section 2). For a perfectly spherical cluster, i.e., all directional vectors of colloids belonging to the cluster point towards the center of the cluster, we have M=1. If clusters have M≥0.9, we consider them as spherical (marked with a red filled circle). Otherwise, if any cluster has 0.5≤M<0.9, we consider it to be a non-spherical cluster (elongated cluster) and mark it with a blue filled square. Finally, clusters of M<0.5 are considered as having randomly oriented patches (depicted by a black asterisk).

**Figure 6:**
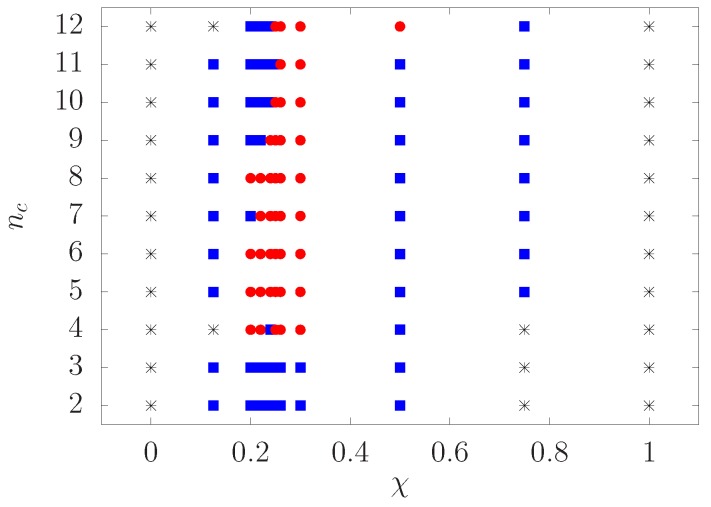
State diagram of the colloidal clusters in the surface coverage-cluster size plane. The symbols indicate the following states of the clusters based on the orientational order parameter: 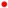, spherical cluster; 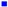, non-spherical cluster; *, clusters with randomly oriented patches.

Using the orientational order parameter of the clusters, we map out the state diagram in the surface coverage *χ*-cluster size $n_{c}$ representation, as shown in Figure 6. We find a narrow region of *χ* in the range 0.20–0.30 in which spherical clusters are favored, and a broader region of non-spherical clusters when *χ* is outside of this range. The clusters of randomly oriented patches are found when *χ* is larger than 0.8 or smaller than 0.2. Of the cluster states observed, the spherical and non-spherical clusters regarded as micelles are interesting. Additionally, the relatively small range of *χ* (0.20–0.30) generating spherical clusters are also in accordance with the values of *χ* that exhibit two isomeric structures, as discussed above. Comparison of M for these two isomers with the same $n_{c}$ (see Table 1) shows that M of $M_{2}$-nonminimal isomers is slightly larger than that of $M_{2}$-minimal isomers (χ=0.25), demonstrating that the former structure has a more spherical shape.

## 4. Conclusions

We have investigated the hierarchical assembly of patchy colloids via emulsion droplet evaporation by means of Metropolis-based kinetic Monte Carlo simulations. We employed the one-patch Kern–Frenkel potential as a generic model for the anisotropic, short-ranged interaction between colloids. The advantage of the Kern–Frenkel potential is that it interpolates smoothly between the isotropic square-well and hard-core potential upon decreasing the attractive coverage *χ* or, equivalently, the bonding angle.

We found that for the cases when χ⩾0.3, our model reproduces $M_{2}$-minimal cluster structures that have been widely observed in both experiments and simulations based on evaporation-induced assembly of colloidal particles. At values of *χ* below 0.3, several additional isomeric structures are produced, including the square dipyramid ($n_{c}=5$), augmented triangular prism ($n_{c}=7$), and square antiprism ($n_{c}=8$). Surprisingly, these structures, which are not members of the $M_{2}$-minimal packings, were also frequently found in experiments. For higher order clusters, we obtain new cluster configurations with the sphenocorona ($n_{c}=10$) and augmented sphenocorona ($n_{c}=11$) shape. In particular, we found that, in all cases in which $M_{2}$-minimal clusters have formed, they must proceed through the packing process of $M_{2}$-nonminimal clusters. In other words, the $M_{2}$-minimal clusters represent collapsed states of $M_{2}$-nonminimal clusters. A further decrease of *χ* below 0.2 produces more isomers with smaller bond-numbers as a direct result of the increasingly difficult equilibration of cluster structures. We note that although the patchy interaction in our model differs from a dipole–dipole interaction between colloidal particles trapped at the droplet surface, our model reproduces most known experimental structures [40]. Therefore, the strongly anisotropic interaction may be a reason for the formation of $M_{2}$-nonminimal cluster structures.

An orientational order parameter M was used to classify the clusters obtained. We found that spherical clusters are only observed in a narrow region of *χ* (0.2–0.3). In addition, for the same number of constituent colloids, the order parameter of $M_{2}$-nonminimal clusters is higher than that of $M_{2}$-minimal clusters. It is therefore plausible to suppose that maximization of the order parameter appears to favor structures that are more spherical.

## Figures and Tables

**Table 1:** Orientational order parameter M for clusters composed of $n_{c}$ colloids and $n_{b}$ bonds for χ=0.25.

$n_{c}$	5	5	7	7	10	10
$n_{b}$	8	9	13	16	22	24
M	0.957	0.934	0.981	0.976	0.935	0.900
